# Bioaccumulation and biological effects of cigarette litter in marine worms

**DOI:** 10.1038/srep14119

**Published:** 2015-09-15

**Authors:** Stephanie L. Wright, Darren Rowe, Malcolm J. Reid, Kevin V. Thomas, Tamara S. Galloway

**Affiliations:** 1 Biosciences, College of Life and Environmental Sciences, University of Exeter, Exeter, Devon EX4 4QD, UK; 2 Norwegian Institute for Water Research (NIVA), Gaustadalléen 21, N-0349 Oslo, Norway

## Abstract

Marine debris is a global environmental issue. Smoked cigarette filters are the predominant coastal litter item; 4.5 trillion are littered annually, presenting a source of bioplastic microfibres (cellulose acetate) and harmful toxicants to marine environments. Despite the human health risks associated with smoking, little is known of the hazards cigarette filters present to marine life. Here we studied the impacts of smoked cigarette filter toxicants and microfibres on the polychaete worm *Hediste diversicolor* (ragworm), a widespread inhabitant of coastal sediments. Ragworms exposed to smoked cigarette filter toxicants in seawater at concentrations 60 fold lower than those reported for urban run-off exhibited significantly longer burrowing times, >30% weight loss, and >2-fold increase in DNA damage compared to ragworms maintained in control conditions. In contrast, ragworms exposed to smoked cigarette filter microfibres in marine sediment showed no significant effects. Bioconcentration factors for nicotine were 500 fold higher from seawater than from sediment. Our results illustrate the vulnerability of organisms in the water column to smoking debris and associated toxicants, and highlight the risks posed by smoked cigarette filter debris to aquatic life.

Marine debris is a global conservation issue^1^. Semi-synthetic bioplastic (rayon) and plastic materials are widely reported in the marine environment^2^. Environmental exposure causes these materials to degrade and fragment, resulting in micron-sized particles and fibres <1 mm (e.g. microplastics)^3^. Fibres are the most frequently reported type of particulate debris, not just in coastal ecosystems, but in deep ocean sediments where recent estimates suggest over 2 billion rayon fibres km^2^ contaminate the seabed^2^.

Smoked cigarette filters – the predominant item reported globally in coastal litter surveys – present a substantial source of rayon microfibres; each filter is comprised of >15,000 cellulose acetate (rayon) fibres, 20 μm in diameter^4^,^5^. Approximately 4.5 trillion smoked cigarette filters, equivalent to >750,000 tonnes, are littered to the environment annually^4^. Despite the anti-littering laws operative in many countries, enforcement at the individual-level is impractical and has proved ineffective in preventing this debris from accumulating in the environment^4^.

Smoked cigarette filters can cause harm in the marine environment in several ways. They present a vector for the transport and introduction of toxicants, including heavy metals, nicotine and known carcinogens^6^, to aquatic habitats. Exposure to such toxicants in seawater could occur following the dissolution of compounds from the bioplastic filter to the surrounding seawater (leaching). Dietary exposure could occur through the ingestion of smoked cigarette filter microfibers due to filter degradation. If ingested, there is potential for the transfer of adhered toxicants to tissues. These bioplastic microfibres and their associated toxicants may persist in the marine environment and continue leaching chemicals for up to 10 years^4^. Despite this, few studies have assessed their potential toxicity. This is particularly important in coastal sediments, where smoked cigarette filters dominate litter^7^,^8^.

Sediment is a vital component of the marine environment, forming one the largest habitats on Earth. Its diverse residents are fundamental to marine ecosystem function, impacting water column processes; trophic transfer; and global biogeochemical cycles^9^. Polychaete worms are widespread and abundant inhabitants of coastal sediments, where they rework and irrigate sediment and form a key prey species for birds and fish^10^,^11^. They adopt a range of feeding strategies, including surface deposit feeding^11^,^12^, and are thus vulnerable to smoked cigarette filter debris and toxicants via both oral and dermal exposure pathways.

For the first time we explore the impacts of smoked cigarette filter toxicants and microfibres on the polychaete worm *Hediste diversicolor* (ragworm). We address the hypotheses that 1) exposure to toxicants desorbed from smoked cigarette filters affects the behaviour and physiology of ragworms, and that 2) smoked cigarette filter microfibres present a physical hazard and/or vector for these associated toxicants. We measure this by quantifying the relative growth rate, burrowing time and level of DNA damage in ragworms exposed to smoked cigarette filter toxicants in seawater or microfibres in sediment, in relation to the bioaccumulation of the biomarker nicotine and its metabolite cotinine.

## Results

### Nicotine Bioaccumulation

**Figure 1:**
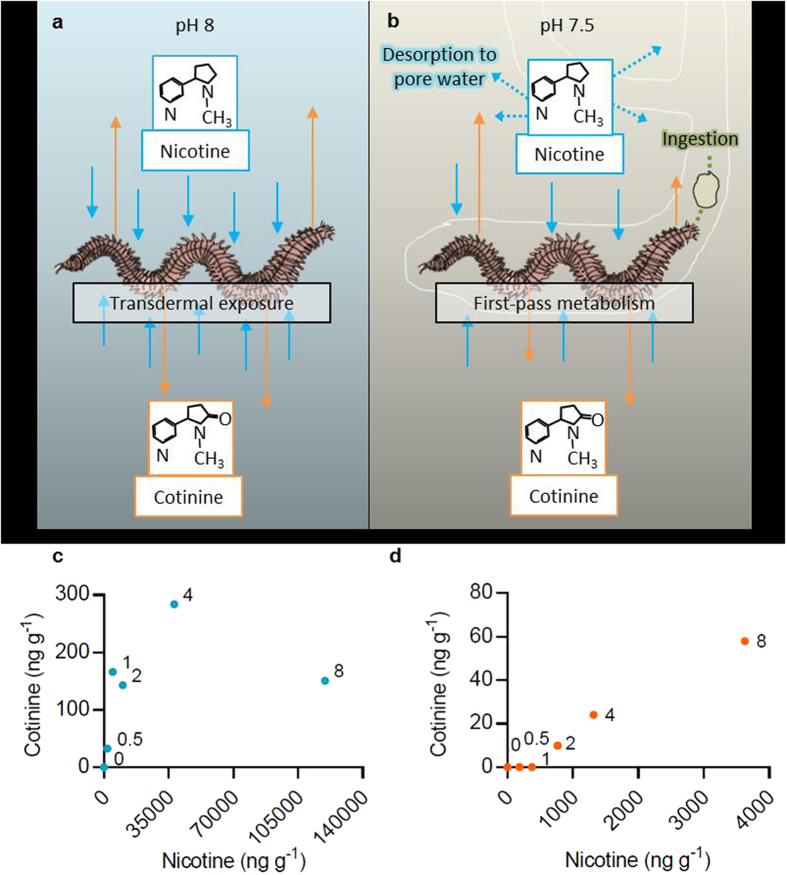
The bioaccumulation of nicotine in ragworms. The potential routes of nicotine transfer to ragworms from smoked cigarette filter (**a**) toxicants in seawater, and (**b**) microfibres in sediment. The bioconcentration of nicotine and cotinine by ragworms following 96 h exposure to smoked cigarette filter (**c**) toxicants in seawater, and (**d**) microfibres in sediment.

Nicotine and its metabolite cotinine were used as biomarkers of exposure to the toxicants associated with smoked cigarette filters (from here on referred to as filters). Nicotine was detected in whole-ragworm tissue following all exposures (see Table 1). After 96 h, the greatest levels of nicotine were measured in ragworms exposed to the highest concentrations of both filter toxicants in seawater (119,654 ng g^−1^ tissue, Bioconcentration Factor (BCF_aqu_) of 172.4) and microfibres in sediment (3,629 ng g^−1^ tissue, Bioconcentration Factor (BCF_sed_) of 0.338) (see Table 1 and Fig. 1c,d). Ragworms accumulated several orders of magnitude less nicotine following both short- and long-term (854 ng g^−1^ tissue, BCF_sed_ of 0.123) sediment exposures to filter microfibres than following exposure to filter toxicants in seawater (Fig. 1d and Supplementary Fig. 2a). The average cigarette contains 0.8–1.9 mg of nicotine. For comparison, this delivers a human dose when smoked of 10–30 μg kg^−1^ based on an average adult weight of 68 kg, resulting in average peak plasma levels of 10–50 ng ml^−1^ 13 (see Table 2).

### Nicotine Metabolism

The nicotine metabolite cotinine was detected in all ragworms following exposure to filter toxicants in seawater (Fig. 1c). Nicotine:cotinine ratios of worm tissues dramatically increased with filter concentration; worms exposed to 8 filters L^−1^ had the greatest ratio (792, see Table 1). Following a 96 h sediment exposure to filter microfibres, cotinine was detected in ragworms exposed to 2 filters L^−1^ and above (Fig. 1d). The greatest nicotine:cotinine ratio was measured in ragworms exposed to 2 filters L^−1^ (76.6, Table 1). After 28 d in sediment, cotinine was detected in ragworms exposed to 4 filters L^−1^ and above (see Table 2 and Supplementary Fig. 2a). The nicotine:cotinine ratio was 67.2 and 61, for 4 and 8 filters L^−1^, respectively. These are similar to the ratios observed in worms exposed to microfibres in sediment over 96 h. These ratios indicate a reduced bioavailability of nicotine via microfibres in the sediment in comparison to filter toxicants in seawater.

### Biological Endpoints

#### Relative Growth Rate

**Figure 2:**
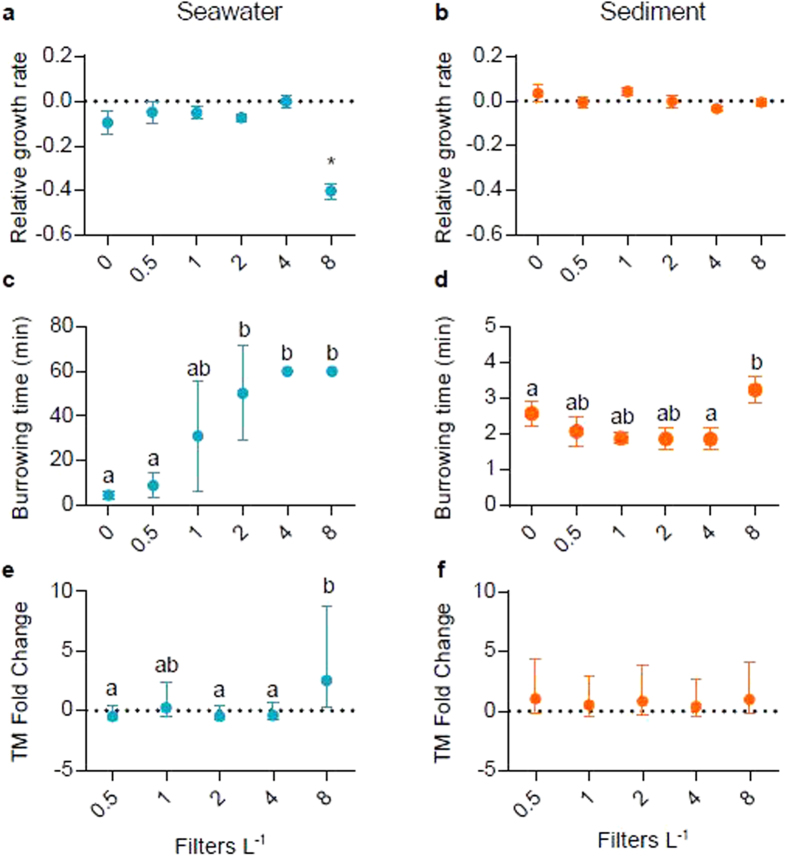
The biological impacts of smoked cigarette filter exposure on ragworms. The effect of 96 h exposure to smoked cigarette filter (**a**) toxicants in seawater, and (**b**) microfibres in sediment on the relative growth rate (RGR) of ragworms (mean ± s.e.m.). The effect of 96 h exposure to smoked cigarette filter (**d**) toxicants in seawater, and (**e**) microfibres in sediment on the burrowing time (minutes) of ragworms (mean ± s.e.m.). The effect of 96 h exposure to smoked cigarette filter (**g**) toxicants in seawater, and h) microfibres in sediment on DNA damage in ragworms, measured as fold-change in the 90^th^ percentile tail moment (TM) relative to control ragworms (indicated by the dotted line, mean ± s.e.m.). Significance between groups, as identified by post-hoc analysis, is indicated by different letters. *denotes significance compared to all other groups.

Relative Growth Rate (RGR) was measured as a general health indicator. A significant effect on RGR was observed in ragworms following exposure to filter toxicants in seawater (one-way ANOVA, p = 0.00005, Fig. 2a). The lowest concentration to cause a significant effect (LOEC) on RGR was 8 filters L^−1^ (−33% mean weight ± 2% s.e.m.). Following 96 h and 28 d sediment exposures to filter microfibres, no effect on the RGRs of ragworms was observed (Fig. 2b and Supplementary Fig. 2b, respectively).

#### Burrowing Activity

Given the neurotoxicity of nicotine^13^, we selected burrowing time as a primary sublethal endpoint. Exposure to the two highest concentrations of filter toxicants in seawater (4 and 8 filters L^−1^) inhibited the burrowing capacity of 100% of individuals during the assay observation period (Fig. 2c). The LOEC for the burrowing time of ragworms exposed to filter toxicants in seawater was 2 filters L^−1^ (Kruskal Wallis, p = 0.0001).

Following a 96 h sediment exposure to filter microfibres, the LOEC for burrowing time was 8 filters L^−1^ (one-way ANOVA, p = 0.04, Fig. 2d). Post hoc analysis showed that this result was significant at a confidence level of 0.1 (Tukey HSD Test, p = 0.07). The burrowing time of ragworms following 28 d sediment exposure to filter microfibres was not affected (Supplementary Fig. 2c).

#### DNA Damage

Exposure to filter toxicants in seawater significantly affected the median, 75^th^, and 90^th^ percentile tail moment (TM, a measure of DNA fragmentation, see Methods) of ragworms (one-way ANOVA, p = 0.016, p = 0.003, and p = 0.003, respectively). Ragworms exposed to 8 filters L^−1^ had significantly greater TMs than those exposed to 0.5, 2, and 4 filters L^−1^ (Fig. 2e, Supplementary Fig. 3a and d for 90^th^, median, and 75^th^ percentiles, respectively). The 75^th^ and 90^th^ percentile tail intensities (TI, a measure of the relative fraction of DNA, see Methods) were also significantly greater in ragworms exposed to 8 filters L^−1^ than to ragworms exposed to 0.5 and 4 filters L^−1^ (Kruskal Wallis, p = 0.04 and p = 0.01, respectively; Supplementary Fig. 4a and d for 75^th^ and 90^th^ percentiles, respectively). Following 96 h and 28 d exposures to filter microfibres in sediment, there was no significant DNA damage (see Fig. 2f, Supplemetary Fig. 3 and 4 b and e; and Supplementary Fig. 2d and 3 and 4 c and f, for 96 h and 28 d, respectively).

## Discussion

This is the first study to assess the impacts of smoked cigarette filter (from here on referred to as filters) debris on a marine invertebrate. We found that exposure to leached filter toxicants in seawater at a concentration of ≥2 filters L^−1^ (172 μg L^−1^ nicotine) significantly inhibited burrowing behaviour in a marine worm, whilst greater concentrations led to reduced growth rates and increased DNA damage. Of the few studies that have assessed the impacts of filter toxicants on aquatic species, water fleas and juvenile fish exhibited greater sensitivity than ragworms did in the present study^14^,^15^. Further investigation is therefore required to determine the impacts of filters on other biotic components of coastal and marine ecosystems.

Ragworms accumulated considerably less nicotine – an established biomarker of exposure to the toxicants associated with smoking - following sediment exposure to filter microfibres than following exposure to filter toxicants in seawater. Notably, the nicotine dose delivered by just one filter L^−1^ via seawater is around 98 times that delivered to a human via smoking (Table 1). Since ragworms were not fed during exposure to filter toxicants in seawater, uptake is anticipated to primarily occur via the epidermis (Fig. 1a). Nicotine is unionized and bioavailable under alkaline conditions^16^. The alkalinity of the seawater in this study (pH 8.06 mean ± 0.007 s.e.m.) indicates over 70% of nicotine was bioavailable, allowing for rapid systemic circulation^16^.

Sediment exposure to filter microfibres and associated toxicants occurs predominantly via indiscriminate surface-deposit feeding. Post-ingestion, up to 70% of nicotine is metabolised before entering systemic circulation^13^ (Fig. 1b). The pH of the sediment measured during low tide (7.5 mean ± 0.01 s.e.m., n = 12) suggests that over 90% of the nicotine is bioavailable in sediment exposures^16^. However, the moderately acidic gut conditions of ragworms could counter this^17^. These factors may explain the low concentration of nicotine detected in ragworms following sediment exposures. Additionally, ragworms are unlikely to encounter the entire sediment volume, thus contacting only a fraction of the contaminant. The worms’ mucus-lined burrow may also act as a physical barrier, limiting encounter rates with nicotine (Fig. 1b). Following 96 h exposure to filter toxicants in seawater, the nicotine:cotinine ratio of ragworm tissue dramatically increased with increasing filter concentration, suggesting metabolism becomes impaired. Nicotine metabolism is important in reducing toxicity: cotinine has a similar mechanism of action to nicotine, but binds to neuronal acetylcholine receptors with lower potency^18^.

If ingested, there is potential for microplastic and bioplastic debris to transfer adhered pollutants, which can accumulate on their surface up to several orders of magnitude greater than the surrounding water column^19^,^20^. Whilst sediment exposure to filter microfibres limited nicotine bioaccumulation, other types of particulate debris have been shown to transport chemical contaminants to invertebrates: microscopic polyvinylchloride (PVC) transferred adhered triclosan and nonylphenol to the gut tissue of sediment-dwelling lugworms, at levels which caused biological harm^21^. Moreover, simulated gut conditions elicited greater desorption rates of chemical contaminants from microscopic polyethylene and PVC than seawater^22^. These studies employed higher concentrations of particles than the current study.

Using the biomarker nicotine, we have shown that filters can act as a vector for the transport and introduction of associated toxicants to seawater through leaching. This may pose an ecological risk to species which could encounter and bioaccumulate these toxicants from the surrounding seawater. However, the ingestion of filter microfibers within sediment by benthic species as a route of exposure to associated toxicants is considered a lower threat.

We show that exposure to filter toxicants in seawater has a significant negative effect on the RGR of ragworms. Similarly, the weight of earthworms was reduced by up to 40% following exposure to the neurotoxic insecticide imidacloprid, which is chemically similar to nicotine^23^,^24^. The authors postulated this was due to decreased feeding, reduced assimilation efficiency, or the up-regulation of an energetically costly detoxification mechanism. Similar modes of toxicity could have also occurred in the present study.

No effect on the RGRs of ragworms was observed following 96 h and 28 d sediment exposures to filter microfibres. The low nutritional value of the cellulose acetate microfibres may be anticipated to reduce RGR. Female rats showed a 14% reduction in growth following prolonged dietary exposure to high doses of cellulose acetate, linked to a nutritional reduction in the feed^25^. The polychaete worm *Arenicola marina* suffered significant reductions in energy reserves following exposure to ≥1% microscopic PVC by weight^26^. This was likely in-part due to a reduction in the nutritional quality of material consumed. The current study employed lower concentrations of microplastics, resulting in a higher proportion of nutritious substrate. We consider the chemical toxicity of leached nicotine and associated toxicants from filters to seawater to be of greater concern than the ingestion of low-nutritive filter microfibers for impacting growth rate.

Exposure to filter toxicants in seawater at a concentration of ≥2 filters L^−1^ significantly affected burrowing activity in ragworms. The insecticide imidacloprid impaired burrowing behaviour in earthworms; burrows were smaller in area and shallower than control groups following a 6 day exposure^27^. Nicotine is neurotoxic, affecting the central and autonomic nervous system and neuromuscular junctions by agonistically binding to the nicotinic acetyl cholinergic receptors (nAChRs)^13^,^28^. This opens ion channels, causing an influx of sodium or calcium ions, increasing the release of neurotransmitters. Prolonged stimulation of nAChRs can lead to desensitization, impairing neurological function^13^. This may explain the inhibited burrowing capacity of ragworms in the current study.

The burrowing behaviour of worms is central to their role as ecosystem engineers, reworking and aerating sediment to allow other organisms to thrive^11^. Nicotine exposure via filter debris presents a potential risk to ecosystem health through its detrimental effects on the burrowing behaviour of worms; this is deserving of further assessment to determine the extent of the risk to the benthic community. As sediment exposure to filter microfibres limited nicotine bioaccumulation, burrowing activity was minimally affected. Filter microfibres within sediment as a vector for nicotine are therefore anticipated to be less neurologically hazardous than filter toxicants in seawater.

An average increase in DNA damage of 2- to 3-fold from control to treatment is considered biologically relevant^29^. A fold increase >2 from control to treatment was observed in the median, 75^th^, and 90^th^ TM percentiles (Fig. 2e, and Supplementary Fig. 3a and d, for median, 75^th^, and 90^th^ percentiles, respectively) and in 75^th^ and 90^th^ TI percentiles (Supplementary Fig. 4a and d, respectively) of ragworms exposed to filter toxicants in seawater. Thus, filter toxicants in seawater at a concentration of 8 filters L^−1^ (694 ng ml^−1^) caused biologically relevant DNA damage, likely due to oxidative stress^30^. However, previous studies have highlighted a protective effect of nicotine on DNA damage at low concentrations through radical scavenging^31^. Ragworms exposed to filter toxicants in seawater at concentrations up to 4 filters L^−1^ (235.5 ng ml^−1^) exhibited significantly lower levels of DNA damage than those exposed to 8 filters L^−1^. This indicates that ragworms experienced the protective effect of low nicotine dosage. At lower nicotine doses, the neurotoxicity of nicotine may be of greater concern than potential DNA damaging effects.

In conclusion, filter toxicants in seawater caused adverse dose-dependent effects on behaviour and high concentrations of filter toxicants effected growth in ragworms, which were linked to nicotine bioconcentration. The concentration of nicotine in the aquatic environment is variable; up to 32 μg L^−1^ in effluent and 11,400 μg L^−1^ in urban run-off have been reported^32^,^33^. It was recently estimated that one smoked cigarette filter could contaminate 1000 L of water at a concentration exceeding the predicted no effect concentration (24 μg L^−1^)^33^. Reported urban run-off concentrations are over 60 times greater than the effective concentration of nicotine in the current study (≥172 μg L^−1^/2 filters L^−1^). Therefore aquatic species in proximity to urbanised areas are at risk of nicotine exposure via run-off contaminated with smoked cigarette filters and their leachates. In comparison, sediment exposure to filter microfibres – an anticipated route of exposure for ragworms in the marine environment - limits the bioaccumulation and toxicity of nicotine. Up to 3.5 cigarette filters m^−2^ has been reported on beaches^34^. Particulate smoked cigarette filter debris is therefore predicted to be of lower risk than leachates. However, it is unknown how the aging of filters and their microfibres would affect nicotine bioaccumulation and toxicity. The quantification of filters in coastal environments as well as the role of aging on filter toxicity are areas deserving of further research.

The protection, conservation and restoration of marine ecosystems increasingly rely on international legislation to curb anthropogenic impacts. Recently, statutory frameworks such as the European Union Marine Strategy Framework Directive (MSFD) have for the first time stipulated that the properties and quantities of marine litter, including microplastics, should not cause harm to the marine environment (Descriptor 10, MSFD, 2008/56/EC). Quantitative toxicological data is essential for supporting the implementation of such legislation; our results provide a first step towards setting guidance limits to curb smoking-related bioplastic debris. We encourage further research into the role of environmental and physiological pH, and different exposure pathways when considering the impacts of filter toxicants and bioplastic microfibers on biotic components of marine ecosystems. Research into the impacts of smoked cigarette filters on marine life is crucial for consolidating the evidence base for remedial policy^4^.

## Methods

### Materials

#### Smoked Cigarette Filters

Smoked cigarette filters (‘filters’, nicotine content 0.7–0.9 mg) were collected and immediately kept in sealed falcon tubes in the dark. Before use, the outer paper and any excess tobacco was carefully removed. Filters were individually weighed to calculate an average filter weight.

#### Chemicals and solutions

Ethyl acetate (Chromasolv HPLC Grade, Sigma Aldrich), methanol (HPLC Super Gradient Reagent, VWR Chemicals), carbon dioxide (food grade, AGA), ammonium hydroxide (ACS Reagent, Sigma Aldrich), AOAC Method 2007.01 Extraction salts (DisQuE, Waters Corp, Milford USA), AOAC Method 2007.01 clean-up tubes (DisQuE, Waters Corp, Milford USA), nicotine, nicotine-D4, cotinine and cotinine-D3 (all from Cerilliant, Round Rock Texas, USA), and 3’-hydroxycotinine (Toronto Research Chemicals, Ontario Canada).

### Exposures and Biological Endpoints

#### Animal Husbandry

The ragworm *Hediste diversicolor* was hand collected from the Exe Estuary, Devon, UK (50°66”76 N, -3°44”40W) between February to April 2014. Stock worms were maintained collectively in 4 cm of natural sediment with overlying artificial seawater (ASW, salinity of 22) in a temperature-controlled room (12 °C, 12 h light:12 h dark). Ragworms were acclimated for at least 1 week. Water changes were performed on alternate days. In all exposures, only healthy, complete ragworms were used.

#### Exposure to Smoked Cigarette Filter Toxicants in Sediment

To establish whether the toxicants associated with filters are harmful to ragworms, an initial aqueous exposure was performed following^14^ and^15^. To produce increasing doses of leachates based on a filter L^−1^ concentration, a leachate stock was produced, also forming the highest concentration (8 filters L^−1^). Smoked filters were placed in artificial seawater (salinity of 22) on an orbital shaker in a temperature-controlled room for 24 h. The leachates were then vacuum-filtered through Whatman cellulose filter paper (grade 1) to remove any particulates due to cigarette filter degradation. The remaining test concentrations were made by performing 0.5x dilution series with the filtered leachate and artificial seawater, achieving final concentrations of 8, 4, 2, 1 and 0.5 cigarette filters L^−1^. Subsamples of each stock concentration were kept at −80 °C for chemical analysis.

Three hundred mL aliquots of leachates were added to 400 mL glass beakers (acid-washed, 13% HNO_3_) immediately before the addition of ragworms. Beakers were randomly allocated a position in a temperature-controlled room (12 °C). Each beaker contained a length of silicon tubing, providing refuge. Beakers were gently aerated and covered to minimise evaporation. Ragworms were weighed and individually transferred to a beaker (n = 6 per treatment group). Observations were made daily. Following 48 h, a water change was performed using fresh leachate from which subsamples were again taken for chemical analysis. Water parameters (salinity, pH, dissolved oxygen) were monitored throughout the exposure period. After 96 h, ragworms were removed from exposure.

#### Exposure to Smoked Cigarette Filter Microfibres in Sediment

To determine whether particulate debris from filters can transfer toxicants at levels capable of causing harm, the impacts of filter microfibers on ragworms were assessed. Filters free of outer paper and excess tobacco were ground under liquid nitrogen using a pestle and mortar until a fine powder formed. Subsamples were suspended in deionised water and observed under a microscope fitted with a camera for size analysis. Individual microfibres were randomly sized using image analysis software.

Microfibers (mean length 120.6 ± 5.1 μm s.e.m., median length 96.5 μm, Supplementary Fig. 1) were added to sediment in bulk by weights equivalent to the concentrations above (number of filters L^−1^). The sediment was manually homogenised. Subsamples of each sediment stock concentration were kept at −80 °C for chemical analysis. Forty eight hours prior to exposures, 225 mL of test sediment was added to 400 mL acid-washed, glass beakers (4 cm depth). Beakers were randomly allocated a position in a temperature-controlled room (12 °C), covered and left to acclimate overnight. Twenty four hours prior to exposures, 150 mL of artificial seawater (salinity of 22) was poured into beakers over a clean, stainless steel spoon. Gentle aeration was provided and beakers were left covered.

Ragworms were weighed and individually transferred to a beaker (n = 6 per treatment group). Observations were made daily and water parameters were monitored throughout the exposure period. Two exposures were conducted, lasting 96 h and 28 d. During the 96 h exposure, a water change was performed after 48 h using fresh ASW. After 96 h, ragworms were removed from the exposure. During the 28 d exposure, water changes were performed every 72 h. Ragworms were not fed during this time as it was assumed they were surface-deposit-feeding on the test sediment. After 28 days, ragworms were removed from exposure. Following endpoint measurements, ragworms were individually maintained in seawater (salinity of 22) to void gut content in preparation for chemical analyses. After approximately 10 h, ragworms were snap-frozen and stored at −80 °C until use.

#### Relative Growth Rate

In addition to pre-exposure wet weights, post-exposure weights were also recorded. Following sediment exposure, any external sediment was carefully rinsed from ragworms. Excess seawater was gently absorbed using a paper towel and ragworms were weighed to 0.01 g.

#### Burrowing activity

Individuals were transferred to 400 mL glass beakers containing 225 mL wet control sediment (corresponding to approximately 4 cm depth). Their burrowing time into clean sediment – from the moment their anterior end touched the sediment to being completely burrowed –was recorded within a 1 h observation period. The burrowing time of ragworms which did not burrow during this time was considered as 60 min.

#### Comet assay

DNA damage – measured as single-strand breaks in individual cells (Comet assay) – was quantified to assess potential carcinogenic and pro-oxidative effects, anticipated due to the constituent toxicants of smoked cigarettes. The Comet assay quantifies DNA damage as tail intensity (TI) and tail moment (TM) for individual cells. TI indicates the relative fraction of damaged DNA. TM is the product of TI and tail length (the migratory distance of broken DNA fragments from the nucleus of the cell), providing a descriptive assessment of DNA damage^35^.

Ragworms were recovered from the burrowing assay and carefully rinsed. A sample of coelomic fluid was withdrawn with a 1 mL syringe containing chilled PBS at a 1:1 ratio, fitted with a 23 gauge needle. Samples were taken from the posterior region, taking care to avoid the gut, and stored on ice until use. One hundred μL of sample was used per individual. Coelomic fluid was centrifuged at 1000 rpm for 3 min and the supernatant was discarded. The cell concentrate was then suspended in 1% low melting point agarose (37 °C) and two aliquots were dropped onto a slide pre-coated with 1% normal melting point agarose. Coverslips were placed on top of the sample and slides were left for 10 min at 4 °C. Once the gel was set, coverslips were carefully removed and the comet assay was conducted, following^36^, modifying for alkaline conditions. Slides were placed in lysis solution for 1 hour, followed by 40 min denaturation in electrophoresis buffer (pH 13) and then electrophoresis for 30 min (25 V, 300 mA). The slides were then gently washed in neutralising buffer. 100 cells per slide (50 per gel) were scored within 48 h using sybr safe staining and a fluorescent microscope (420–490 nm excitation filter and 520 nm emission filter) equipped with Kinetic COMET software.

### Chemical Analysis

Nicotine and its metabolite cotinine were used as biomarkers of exposure to the toxicants associated with filters. Frozen ragworm tissue was thoroughly homogenised under liquid nitrogen using a pestle and mortar. For each exposure and concentration sub-aliquots of homogenised tissue from each individual were pooled.

#### Chromatography and detection (MS/MS) parameters

Analysis was carried out on an Acquity UPC2 system with a Quattro Premier XE Mass Spectrometer (MSMS) as detector (both from Waters Corp, Milford USA). See Supplementary Table 1 for details.

#### Sample Preparation

Water Samples: Five hundred μL samples of aqueous exposure media (water) were spiked with internal standard solution (25 μL of a solution containing 500 ng/mL nicotine-D4 and cotinine-D3) and then adjusted to pH 10 with ammonia. Liquid-liquid extraction was performed with 1 mL ethyl acetate. The upper (ethyl acetate) phase was removed and analysed.

Sediment Samples: Sub-samples (0.5 g) were weighed into 10 mL glass test-tubes and spiked with internal standard solution (100 μL of a solution containing 500 ng/mL nicotine-D4 and cotinine-D3) together with 3 mL water (2% ammonium hydroxide) and 4 mL acetonitrile. Samples were then extracted and cleaned according to AOAC Method 2007.01 for pesticide residues in foods by acetonitrile extraction and partitioning with magnesium sulphate (REFERENCE: available online at http://www.eoma.aoac.org/methods/info.asp?ID=48938).

Ragworm Samples: Fifty milligram ragworm samples were weighed into 2 mL tubes and spiked with internal standard solution (10 μL of a solution containing 500 ng/mL nicotine-D4 and cotinine-D3) together with 300 μL water (2% ammonium hydroxide) and 400 μL acetonitrile. Samples were then extracted and cleaned according to AOAC Method 2007.01 for pesticide residues in foods by acetonitrile extraction and partitioning with magnesium sulphate (REFERENCE: available online at http://www.eoma.aoac.org/methods/info.asp?ID=48938).

### Statistical Analyses

Statistical analyses were performed in R^37^. To ensure correct specification of the models used (analysis of variance), the distribution of residuals was monitored using the Shapiro Wilks test for normality and Levene’s test for homogeneity of variance. Where data did not conform to model assumptions, a log_10_(*x* + 1) transformation was performed. If this did not increase suitability, an equivalent non-parametric test was performed.

Any change in the weight of ragworms during exposures was assessed using the method of^38^. First, relative growth rate (RGR) was calculated as in equation 1:


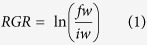


where *fw* = final weight and *iw* = initial weight. The effect of treatment on RGR was then analysed using a one-way ANOVA (n = 6).

Any change in burrowing time due to exposure was determined using a one-way ANOVA or Kruskal Wallis test where appropriate (n = 6). A change in tail intensity (TI) and tail moment (TM) was analysed using the methods of^29^,^39^,^40^, whereby the statistical analysis is performed by animal (as opposed to by gel or by cell) using a summary statistic calculated by equation 2:


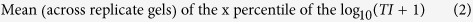


where x is substituted for the median, 75^th^, or 90^th^ percentile based on recommendations by^29^,^40^. The effect of treatment on TI and TM was then analysed using a one-way ANOVA or Kruskal Wallis test (n = 6).

Where a Kruskal Wallis was applied and a significant p-value obtained, post-hoc Wilcoxon rank sum tests were used on pairwise permutations. Post-hoc analysis following a one-way ANOVA was conducted using a Tukey HSD test. The lowest concentration which elicited a significantly different response compared to the control was identified as the Lowest Observed Effect Concentration (LOEC), whilst the highest concentration which did not cause a significantly different response compared to the control was identified as the No Observed Effect Concentration (NOEC).

#### Bioaccumulation

Bioconcentration factors (BCF_aqu_ and BCF _sed_) – the level of accumulation of a chemical in an organism from seawater and sediment, respectively - were calculated. This was quantified using the following calculation (equation 3):


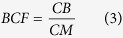


Where *CB* = biota concentration and *CM* = medium (leachate or sediment) concentration.

## Additional Information

**How to cite this article**: Wright, S. L. *et al.* Bioaccumulation and biological effects of cigarette litter in marine worms. *Sci. Rep.* **5**, 14119; doi: 10.1038/srep14119 (2015).

## Supplementary Material

Supplementary Information

## Figures and Tables

**Table 1:** Nicotine concentrations measured in the stock exposure medium and whole ragworm tissue following 96 h exposure to smoked cigarette filter toxicants in seawater, and 96 h and 28 d exposure to smoked cigarette filter microfibres in sediment.

**Filters L**^**−1**^	**Nicotine (ng ml^−1^, ng g^−1^)**			**Nicotine (ng g^−1^ tissue )**			**BCF_aqu_, BCF_sed_**			**Nicotine:Cotinine ratio**		
	**Leachates**	**Sediment**		**Leachates**	**Sediment**		**Leachates**	**Sediment**		**Leachates**	**Sediment**	
	96 h	96 h	28 d	96 h	96 h	28 d	96 h	96 h	28 d	96 h	96 h	28 d
0	5.5	0	0	0	0	0	0	0	0	0	0	0
0.5	23.5	787	350	1901	186	41	80.89	0.24	0.12	57.6	0	0
1	62.5	1399	971	4912	374	129	78.59	0.27	0.13	29.6	0	0
2	172	3124	1759	10193	766	211	59.26	0.25	0.12	71.3	76.6	0
4	235.5	5287	3743	38072	1318	672	161.66	0.25	0.18	134.1	54.9	67.2
8	694	11159	6964	119654	3629	854	172.41	0.33	0.12	792.4	62.6	61

BCF_aqu_ = bioconcentration factor from seawater, BCF_sed_ = bioconcentration factor from sediment.

**Table 2:** The nicotine dose delivered to: a human smoker; a ragworm following 96 h exposure to smoked cigarette filter toxicants in seawater; and a ragworm following short- and long-term exposure to smoked cigarette filter microfibres in sediment, at equivalent concentrations (1 cigarette/filter L^−1^).

**Organism**	**Dose delivered (1 cigarette)**	**Dose accumulated (1 cigarette)**	**Nicotine exposure relative to humans**
Human (smoking; 1 cigarette)	10–30 μg kg^−1^	10–50 ng ml^−1^	—
Ragworm (96 h exposure to smoked cigarette filter toxicants in seawater)	63 μg kg^−1^	4912 ng g^−1^	98×
Ragworm (96 h sediment exposure to smoked cigarette filter microfibres)	1400 μg kg^−1^	374 ng g^−1^	7.5×
Ragworm (28 d sediment exposure to smoked cigarette filter microfibres)	1000 μg kg^−1^	129 ng g^−1^	2.6×
