# Supplementary material for: Bioaccumulation and biological effects of cigarette litter in marine worms
Source: Sci Rep. 2015 Sep 15;5:14119. doi: 10.1038/srep14119 (PMC4569891; doi:10.1038/srep14119)
Supplement: Supplementary Information [file srep14119-s1.doc]

**Supplementary Information: Bioaccumulation and biological effects of cigarette litter in marine worms** Stephanie L. Wright^1^*, Darren Rowe^1^, Malcolm J. Reid^2^, Kevin V. Thomas^2^, and Tamara S. Galloway^1^*

| Supplementary Table 1. Chromatography and MS/MS detection parameters for the analysis of nicotine and metabolites | | |
| --- | --- | --- |
| ***Chromatograhy Parameters*** | | |
| Column | Waters Acquity BEH 2-EP column, 1.7 µm, 3x100 mm, at 50 °C | |
| Mobile phase | (A) Super-critical carbon dioxide  (B) methanol (0.5 % ammonia) | |
| Flow (Back-pressure) | 1.0 mL/min (2000 PSI) | |
| Gradient | Initial 0.5 % phase (B) hold for 0.1 minutes then ramp up to reach 50 % (B) at t = 2.5 minutes. Hold to t = 3.0 minutes then step down to initial conditions at t = 3.01 minutes. Hold at initial conditions until completion. Total gradient program, t = 4.0 minutes. | |
| ***MS/MS Detection Parameters*** | | |
| Compound | Precurser Ion > Fragment Ions (m/z) | Retention Time (mins) |
| Nicotine | 163>106  163>130 | 1.93 |
| Trans-3'-hydroxycotinine | 193>80  193>134 | 2.16 |
| Cotinine | 177>80  177>98 | 2.06 |
| Nicotine-D4 | 167>121  167>134 | 1.93 |
| Cotinine-D3 | 180>80  180>101 | 2.06 |

Supplementary Figure 1. a) The size-frequency distribution of stock smoked cigarette filter microfibers; b) An intact smoked cigarette filter free of excess tobacco and external paper; c) The fibrous nature of cigarette filters; d-e) micrographs of smoked cigarette filter microfibres following manufacture using liquid nitrogen.


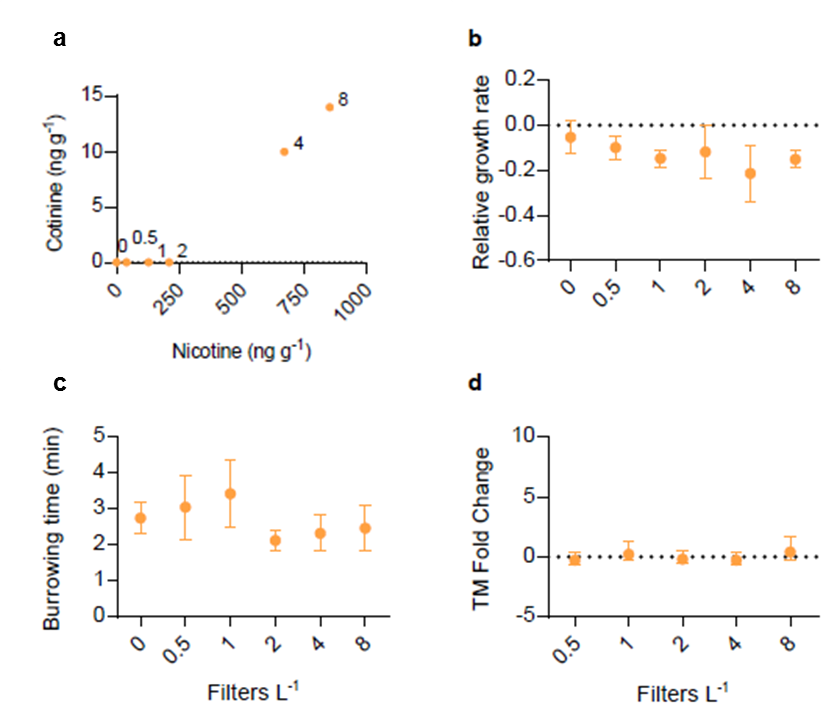


Supplementary Figure 2. The biological impacts of smoked cigarette filter exposure (28 d) on ragworms. a) The bioconcentration and bioaccumulation of nicotine and cotinine for ragworms following 28 d exposure to smoked cigarette filter microfibres in sediment. The effect of 28 d exposure to smoked cigarette filter microfibres in sediment on a); b) the relative growth rate (RGR) of ragworms (mean ± s.e.m.); c) the burrowing time (minutes) of ragworms (mean ± s.e.m.); and d) on DNA damage in ragworms, measured as fold-change in the 90^th^ percentile tail moment relative to control ragworms (indicated by the dotted line, mean ± s.e.m.).

Supplementary Figure 3. The impacts of smoked cigarette filter exposure on DNA damage (tail moment). The effect of 96 h exposure to smoked cigarette filter a) toxicants in seawater, b) microbfibres in sediment, and c) 28 d exposure to microfibres in sediment, on DNA damage in ragworms, measured as fold-change in the median tail moment relative to control ragworms (indicated by the dotted line, mean ± s.e.m.). The effect of 96 h exposure to smoked cigarette filter d) toxicants in seawater, e) microbfibres in sediment, and f) 28 d exposure to microfibres in sediment, on DNA damage in ragworms, measured as fold-change in the 75^th^ percentile tail moment relative to control ragworms (indicated by the dotted line, mean ± s.e.m.). Significance between groups, as identified by post-hoc analysis, is indicated by different letters.

Supplementary Figure 4. The impacts of smoked cigarette filter exposure on DNA damage (tail intensity). The effect of 96 h exposure to smoked cigarette filter a) toxicants in seawater, b) microbfibres in sediment, and c) 28 d exposure to microfibres in sediment, on DNA damage in ragworms, measured as fold-change in the 75^th^ percentile tail intensity relative to control ragworms (indicated by the dotted line, mean ± s.e.m.). The effect of 96 h exposure to smoked cigarette filter d) toxicants in seawater, e) microbfibres in sediment, and f) 28 d exposure to microfibres in sediment, on DNA damage in ragworms, measured as fold-change in the 90^th^ percentile tail intensity relative to control ragworms (indicated by the dotted line, mean ± s.e.m.). Significance between groups, as identified by post-hoc analysis, is indicated by different letters.
